# Sex/Gender Differences in the Language Profiles of Italian Children with Autism Spectrum Disorder: A Retrospective Study

**DOI:** 10.3390/jcm12154923

**Published:** 2023-07-26

**Authors:** Jessica Barsotti, Gloria Mangani, Roberta Nencioli, Antonio Narzisi, Lucia Pfanner, Anna Maria Chilosi, Paola Cipriani, Alice Mancini, Angela Cosenza, Raffaella Tancredi, Sara Calderoni

**Affiliations:** 1 Department of Developmental Neuroscience, IRCCS Stella Maris Foundation, Viale del Tirreno 331, 56128 Pisa, Italy; jessica.barsotti@fsm.unipi.it (J.B.); gloria.mangani@fsm.unipi.it (G.M.); roberta.nencioli@fsm.unipi.it (R.N.); antonio.narzisi@fsm.unipi.it (A.N.); lucia.pfanner@fsm.unipi.it (L.P.); a.chilosi@fsm.unipi.it (A.M.C.); paolacipriani1940@gmail.com (P.C.); alice.mancini@fsm.unipi.it (A.M.); angela.cosenza@fsm.unipi.it (A.C.); raffaella.tancredi@fsm.unipi.it (R.T.); 2 Department of Clinical and Experimental Medicine, University of Pisa, Via Roma 55, 56126 Pisa, Italy

**Keywords:** language and communication, sex/gender, autism spectrum disorder, children

## Abstract

Sex/gender (S/G) differences in ASD language profiles have been poorly investigated. The present study aims to explore whether male (M) and female (F) children with ASD and with normal non-verbal cognitive abilities differ in their linguistic profiles. A sample of 76 Italian children with ASD (range: 4.9–8 years), including 50 Ms and 26 Fs, was retrospectively recruited. Language profiles were analyzed using standardized tests for the evaluation of receptive and expressive vocabulary as well as grammar. Grammatical comprehension was the most impaired domain compared to the other language measures in both M and F children. Comparing language profiles between S/G, Fs showed significantly better scores than Ms in grammatical production (*p* = 0.002), and Ms showed better active negative sentence comprehension (*p* = 0.035). Moreover, comparing the language profiles between Ms and Fs with a receptive disorder, Fs had significantly worse grammatical comprehension and better grammatical production than Ms. Even among children without a receptive disorder, Fs had significantly higher grammatical production scores. The S/G differences in language profile, particularly better expressive language in Fs than Ms, can partially contribute to the delayed ASD diagnosis or underdiagnosis of Fs without intellectual disability. Finally, the results document the importance of accurately investigating both expressive and receptive abilities in children with ASD.

## 1. Introduction

Autism spectrum disorder (ASD) is a neurodevelopmental disorder characterized by persistent deficits in reciprocal social interaction, communication and the presence of restricted, repetitive behaviors and interests [1]. Prevalence data from the most recent study conducted by the American Centers for Disease Control and Prevention reported that ASD occurs in approximately one in every 36 children aged 8 years [2]. In Italy, the prevalence resulting from a cross-sectional epidemiological study conducted in the metropolitan area of Pisa (Tuscany, Central Italy) was 1 in 87 among children aged 7 to 9 years [3].

In the field of typical development, extensive literature reported that Ms and Fs differ in the rates of communication and language development. In particular, Fs demonstrate an earlier acquisition of first words [4], a better and earlier integration of language with gesture [5], and an earlier use of social–emotional words and of more complex linguistic forms during spontaneous speech [6]. Regarding conversational skills, Fs use more collaborative and negotiated discourse [7] and focus on person-centered topics and emotions [8]. These profiles are crucial for social–linguistic interaction and integration with female social groups [8,9]. Conversely, S/G differences in language profiles of children with ASD are still poorly investigated.

According to the fifth edition of the Diagnostic and Statistical Manual of Mental Disorders [1], language impairment is no longer a core symptom of ASD but an ASD ‘specifier’ used for a more detailed description of the patient’s characteristics. Indeed, the level of language skills is highly heterogeneous in ASD, ranging from individuals who never develop spoken language (approximately one-third of subjects, according to Koegel et al. [10]) to individuals with preserved expressive language abilities but with deficits in the pragmatic use of language [11]. Moreover, most toddlers show a delay in standard early language milestones that in some children can be recovered around three or four years of age, while others may show a regression of language after the acquisition of first words at 12–15 months [12].

The over-representation of boys with respect to girls is one of the most replicated findings in the ASD literature [13,14,15]. Indeed, the sex imbalance prevalence in ASD, with an approximate 4:1 male (M) to female (F) ratio [16] that shifted toward a 3:1 ratio in epidemiological screening surveys on the general population [17], has historically impacted the scientific knowledge (clinical, genetic, and neuroanatomical) of ASD in Fs [18]. This topic has been poorly investigated, and only recently, a growing number of studies have focused on it [17].

To note, given the difficulties in disentangling the effects of sex and gender on ASD features, the term “sex/gender” (S/G) will be used throughout this article to acknowledge the overlap between these two concepts [19].

In toddlerhood, language delay is a major cause for concern among parents of infants who go on to receive an ASD diagnosis [20], and this symptom is more prominent in autistic Ms compared to Fs [21]. Moreover, the production of first words and sentences occurs earlier in ASD Fs than in ASD Ms [22]. Indeed, S/G differences in language delay may contribute to the delayed ASD diagnosis in some Fs compared to Ms [23,24] since earlier ASD diagnoses are associated with parent-reported expressive language delays [25]. According to this view, findings from a retrospective investigation reported that ASD Fs, who received a diagnosis of autism after the age of 5 years, displayed more advanced social communication skills, including vocabulary [26]. Vice-versa, autistic Fs receiving a diagnosis during toddlerhood often displayed co-occurring language delays and/or intellectual disability [27].

Controversial findings regarding S/G differences in language and communication domains were reported. Some studies did not find any statistically significant differences in basic vocabulary, grammar skills, and on narrative abilities between Fs and Ms [28,29], whereas others showed S/G differences in pragmatic and narrative abilities [29,30,31,32,33,34,35]. Regarding semantic abilities, Sturrock et al. [29] and Goddard et al. [36] found that ASD Fs performed better than ASD Ms using similar word-generation/fluency tasks.

Moreover, ASD Fs performed better than ASD Ms on narrative skills (including salient storytelling, rich narrative details, and use of internal state language) [33] and on clinical observations of pragmatic abilities [35].

The abovementioned studies focused more on social and pragmatic domains rather than on basic structural language and identified pragmatic and associated higher-level structural language skills as areas of difference between the M and F phenotypes of ASD [29,30,33]. Indeed, S/G differences appear to exist mainly in domains where the meaning of structural language is mediated by social context, inference, language of emotion and internal state, and pragmatic behaviors in discourse and narratives [37].

To address the abovementioned knowledge gap, the present study aimed to investigate S/G differences in basic structural language profiles in a cohort of children with ASD. Specifically, it directly compared expressive and receptive language abilities between M and F Italian children with ASD, using both naturalistic and standardized assessments performed by speech–language therapists with expertise in communication disorders. Since a significant association between non-verbal IQ and language abilities was previously detected [38,39], it was decided to focus the investigation on a homogeneous group of children without intellectual disability.

Based on this previous evidence, we formulated three hypotheses: Hypothesis 1: We expected to find significant phenotypic differences related to S/G language profile; Hypothesis 2: We expected to find differences between ASD Ms versus ASD Fs with receptive language disorder; and Hypothesis 3: We expected to find differences between ASD Ms versus ASD Fs without receptive language disorder.

## 2. Materials and Methods

### 2.1. Sample

The study was conducted on a sample of 76 children with ASD (50 males and 26 females) aged 4.9–8 years (mean age: 6.4 years; SD: 12.1 months) retrospectively recruited in a tertiary care University hospital from February 2009 to November 2020.

Inclusion criteria were as follows: (1) Diagnosis of either autistic disorder according to DSM-IV-TR criteria [40] or autism spectrum disorder according to DSM-5 criteria [1]; (2) Non-verbal IQ or developmental quotient ≥70, as assessed through standardized psychometric tests (i.e., Wechsler scales; Griffiths scales); (3) Expressive language at the level of multi-words production. Exclusion criteria consisted of (1) neurological syndromes or focal neurological signs; (2) birth asphyxia; (3) premature birth before 34 weeks of pregnancy; (4) epilepsy; (5) significant sensory impairment (e.g., blindness, deafness); (6) known monogenic syndromes associated with ASD (e.g., Fragile X Syndrome, Rett Syndrome, and Tuberous Sclerosis Complex).

Informed written consent was obtained from the parents of all participants. This study was approved by the Pediatric Ethical Committee of the Tuscany Region (approval number: 178/2016) and was conducted according to the Helsinki Declaration.

### 2.2. Instruments

The language profile was evaluated by the following measures:

- Grammatical Comprehension Test for Children (TCGB) [41] is standardized on Italian children aged 3.6–8 years. The TCGB is a picture multiple-choice language test composed of 76 sentences pertaining to eight main blocks of grammatical structures (locatives, inflectionals, both affirmative and negative actives and passives, relatives and datives).
- Peabody Picture Vocabulary Test-Revised (PPVT-R) [42] is a multiple-choice language test for receptive vocabulary for 3.9–11.6 years old children.
- One-Word Picture Vocabulary Test [43] is an expressive picture-naming test for high and low-frequency words), for 4.6–10.8 years old children.
- The ‘Grid of Analysis of Spontaneous speech’ (GASS) [44,45] is a grid for the analysis of spontaneous language performed according to six levels rating system based on syntactical and morphological criteria.

For all language tests (with the exception of the GASS), z scores below −1.5 SD of the mean were considered clinically significant. For a detailed description of TCGB and GASS, see Tables S1 and S2 in Supplementary Materials.

WPPSI-III [46] performance IQ or Perceptual Reasoning Index at WISC-IV [47] or Griffiths [48] developmental quotient of the performance scale were used as measures of the non-verbal intellectual functioning level.

ADOS-G [49] or ADOS-2 [50], considering comparison score, was performed with ASD children for the evaluation of autistic severity. All the alpha values of the research tools used are 0.90 and above, except for the One-Word Picture Vocabulary Test, whose value was not reported by the author.

### 2.3. Statistical Analysis

Skewness and Kurtosis statistics did not demonstrate a normal distribution for language-related variables. Thus, non-parametric tests were used.

The Mann–Whitney U test was used to compare age, language scores (z scores for TCGB and One-Word Picture Vocabulary, Lexical Quotient for PPVT-R and GASS level), non-verbal, verbal and total cognitive scores, and ADOS severity scores:

1. between S/G (50 ASD M and 26 ASD F);
2. between ASD M and ASD F with receptive disorder (31 ASD M and 17 ASD F);
3. between ASD M and ASD F without receptive disorder (19 ASD M and 9 ASD F).

Statistical analyses were performed using SPSS 21 software (IBM SPSS Statistics, Chicago, IL, USA).

## 3. Results

Considering the mean z scores of the whole sample (see Table 1), grammatical comprehension (TCGB) was the most impaired domain compared to the other language measures (receptive and expressive vocabulary) in both M and F groups. The TCGB mean total z score and the mean z score of the different language structures (except for active negative sentences in the M group) fell below minus 1.5 SD of the mean (see Table 1). The expressive vocabulary (One-Word Picture Vocabulary Test) was the better linguistic area in both groups (mean z score > 1.5).

The analysis of the number of children who presented deficient expressive grammatical abilities (GASS) showed that there was a higher percentage of Ms (15%) than Fs (4%) with impaired performance. Moreover, receptive vocabulary and grammar were the most deficient areas in a high percentage of children in both the M and the F groups (receptive vocabulary: M = 42%, F = 56%; grammatical comprehension: M = 62%, F = 65%).

Statistical analysis with the Mann–Whitney U test did not show any significant differences in age, cognitive abilities, and severity of autistic symptoms between Ms and Fs (Table 1). Instead, the two groups showed significant differences in expressive grammatical abilities and in active negative sentence comprehension. In particular, Fs showed significantly better scores than Ms in grammatical production (*p* = 0.002), whereas Ms had better active negative sentence comprehension (*p* = 0.035). Moreover, Ms showed a better grammatical comprehension total score than Fs, though this finding did not reach statistical significance.

Given the importance of receptive difficulties, Mann–Whitney U tests have been conducted to compare the functional profiles of Ms and Fs with and without receptive disorder. Both analyses (comparisons between Ms and Fs with and without receptive disorder) did not show any significant differences in non-verbal cognitive scores and severity of autistic symptoms between the groups (Table 2). Both receptive (*p* = 0.014) and expressive (*p* = 0.019) grammatical abilities differed significantly between Ms and Fs in children with receptive deficits. In particular, Fs had significantly worse grammatical comprehension but significantly better grammatical production than Ms.

Statistical comparison between Ms and Fs without receptive disorders also confirmed in this group that Fs had significantly better expressive grammatical abilities (*p* = 0.028) than Ms.

## 4. Discussion

The results of the present study showed that both M and F groups displayed a deficit in grammatical comprehension, and this weakness was more evident in ASD Fs than in ASD Ms. Moreover, in the sample, Fs presented significantly better grammatical production skills than Ms.

These findings thus confirm not only the presence of a marked impairment of receptive skills in children with ASD [51,52,53,54,55,56] but also, especially in the F group, a strong discrepancy between language comprehension and production already documented in the literature [56,57,58]. This discrepancy between grammatical production and comprehension may make it difficult to identify the receptive disorder. In fact, better production skills may mask the comprehension deficits, thus not allowing access to specific rehabilitation interventions.

The above data should be interpreted with caution, as the few papers that addressed this issue report conflicting results. For example, Sturrock et al. [29] compared ASD Ms and Fs with PIQ ≥ 70 and proposed them a battery of direct assessments targeting expressive and receptive language at multiple levels: word, simple and complex sentences, narratives, word knowledge (semantics), inference and vocabulary of emotion. These authors did not find any statistical differences in grammar skills (and basic vocabulary) between Ms and Fs. Moreover, in a recent paper, Sturrock et al. [37] provide a synthesis of recent studies investigating language and communication difficulties in autistic Fs without intellectual disability. The authors found that autistic Fs appeared to perform below typically developing Fs on measures of pragmatics, semantics, and above sentence-level structural language; however, vocabulary and basic grammar (receptive and expressive) appeared to be unaffected. These data are consistent with the review of McFayden and colleagues [59], in which S/G differences are most evident when social communication is evaluated in a naturalistic context rather than based on a standardized assessment. In fact, parents of Fs described greater difficulties than those detected through direct standardized measurement [60,61].

Conversely, the results of the present study are in line with some works detecting better performance of Fs than Ms in some expressive language tasks. In this context, Sturrock et al. [29] and Goddard et al. [36] found that autistic Fs performed better than autistic Ms using similar word-generation/fluency tasks.

Other studies demonstrated S/G differences in pragmatic elements of narratives, with autistic Fs generating richer character depictions and descriptions of internal states, cognition, perception, and judgment [30,32,33] and overall better skills in retelling salient story elements [33]. For instance, autistic girls used significantly more social words than autistic boys during the conversation section of the ADOS-2 Module 3 [62].

All these data, documenting the best expressive language and pragmatic abilities, including social words used and grammatical production (as found in the present study), can partially contribute to the undiagnosed or late-diagnosed ASD in Fs. Moreover, the specific linguistic profile of high-functioning Fs with ASD has been related to the “camouflage” abilities of these individuals (for a recent review, see Tubío-Fungueiriño et al., [63]). Indeed, the term “camouflaging” is used to describe the strategies, either voluntarily or involuntarily, adopted by ASD subjects for masking or compensating for the social impairment experienced during social interactions [31]. Even if the majority of the investigations on social camouflaging to date focused on F adolescents and adults with ASD, this feature is also present in children with ASD [64,65,66]. Recent research suggests that girls with ASD may “camouflage” real struggles with social communication by engaging in verbal and social communication, social mimicry, and behaving in ways that are superficially typical, and these factors, combined with male-referenced diagnostic criteria and unequal societal expectations for boys’ and girls’ social interaction skills across development, may complicate ASD diagnosis [17,59,67,68,69,70]. Accordingly, a late or inaccurate diagnosis for females with ASD may result in difficulty accessing evidence-based interventions and in a lack of social support. In this way, there is a high risk of experiencing social rejection and poor mental health outcomes [59,71,72].

The results of the present study suggest the presence of distinct linguistic profiles in ASD Ms and Fs with IQ ≥ 70. They also provide evidence of the importance of accurately investigating both expressive and receptive language abilities for the choice of effective and personalized interventions aimed at promoting language development on the basis of the specific language profile.

Moreover, enhancing pediatricians’ understanding of language disorders in ASD is crucial as it could play a pivotal role in achieving early detection and diagnosis. Providing comprehensive information and training to pediatricians could help them to recognize the subtle signs and challenges associated with language impairments in ASD. This, in turn, facilitates timely identification and referral to specialists for further evaluation and intervention. Investing in pediatricians’ knowledge and expertise in this area not only enables early intervention but also improves outcomes for children with ASD, ensuring they receive the necessary support and tailored interventions from an early age.

The present study has certain limitations that must be acknowledged. First, the rather low sample size, the lack of power analysis, and the retrospective nature of this single-center investigation make the study susceptible to bias: therefore, the results obtained need to be further validated on a larger sample of ASD children.

Second, only ASD subjects without an intellectual disability were included. This selection criterion was justified by the need to homogenize the sample of children with ASD, but it does not allow to generalize results to the rest of the ASD population. In order to overcome, at least in part, the above limitation, in future studies, it might be very interesting to compare the Verbal Intelligent quotient to the receptive and expressive verbal skills in M and F groups. In this way, more information about the differences between Ms and Fs with ASD might be collected, thus improving the ability to describe phenotypic variability.

Third, the lack of a matched control group of typically developing children was a limitation of the study but was mitigated by the use of standardized tests.

Fourth, the alpha value of the One-Word Picture Vocabulary Test was not reported.

Future studies should integrate clinical assessments performed by trained professionals with parent-report measures of communication abilities in order to obtain a more comprehensive picture of S/G ASD differences in the use of language in daily living situations.

## 5. Study Implications

The study may have implications in both clinical and research settings.

The clinical implications could concern the following points: (1) Improved Diagnosis: the findings of this study suggested that considering S/G differences in language profiles can contribute to more accurate and timely diagnoses of ASD, especially in females without an intellectual disability. Clinicians can incorporate this knowledge into their assessment process to ensure a comprehensive evaluation of language abilities in both male and female individuals with ASD; (2) Tailored Interventions: Understanding the specific language difficulties experienced by males and females with ASD can inform the development of targeted intervention strategies. Clinicians can design interventions that address the unique language profiles of each S/G, focusing on areas such as grammatical comprehension or production where individuals may struggle the most; (3) Enhanced Support: By recognizing the S/G differences in language profiles, clinicians can provide more effective support to individuals with ASD. Tailored therapy approaches can be implemented to enhance both expressive and receptive language skills, considering the strengths and challenges associated with each S/G.

The research implications could cover the following points: (a) Further Exploration of S/G Differences: The study pointed out the need for more research on S/G differences in language profiles among individuals with ASD. Future studies should investigate larger and more diverse samples to validate and expand upon the findings. Longitudinal studies should also examine how language abilities evolve over time and whether these differences persist into adulthood. Moreover, the study of S/G differences in language profiles would benefit from the inclusion of siblings at high familial risk for ASD in which decreased early language ability has been detected [73]; (b) Underlying Mechanisms: The study opens avenues for exploring the underlying mechanisms that contribute to the observed S/G differences in language profiles. Researchers can investigate genetic, hormonal, neurobiological, or social factors that may play a role in shaping these differences, providing a deeper understanding of the etiology and developmental trajectories of ASD. In this framework, neuroanatomical magnetic resonance imaging (MRI) studies indicated that language impairments are related to atypical lateralization in terms of extreme rightward patterns [74], and functional MRI investigations revealed right hemisphere hyperactivation in crucial language areas such as superior temporal gyrus and inferior frontal gyrus [75]; however, literature is lacking well-powered MRI studies focused on brain underpinnings of linguistic profiles in ASD Ms and Fs separately; (c) *Intervention and Outcomes:* Building on the study’s findings, further research could explore the effectiveness of S/G-specific interventions in improving language skills and overall outcomes for individuals with ASD. Comparative studies could assess the impact of tailored interventions on language development, social communication, and quality of life in males and females with ASD. In summary, the study has implications for clinical practice by influencing diagnostic approaches, intervention strategies, and support provided to individuals with ASD. Moreover, it highlights areas for further research, including investigating underlying mechanisms and exploring S/G-specific interventions and outcomes. These implications contribute to advancing our understanding of ASD and ultimately improving the lives of affected individuals.

## Figures and Tables

**Table 1:** Male and female sample characteristics (*n* = 76) and Mann–Whitney U test.

		Males (*n* = 50)			Females (*n* = 26)		
	Mean	SD	Mean z Score	Mean	SD	Mean z Score	*p*
**Age**	76.08	12.54	-	79.58	12.67	-	ns
**Grammatical Comprehension score (TCGB)**	20.41	12.14	−2.93	21.08	9.59	−4.77	ns
Locative	2.60	2.27	−1.67	2.87	2.07	−1.90	ns
Inflectional	3.45	2.65	−2.36	3.15	2.01	−4.27	ns
Affirmative Active	2.14	2.04	−2.60	2.02	1.43	−4.06	ns
Negative Active *	1.94	1.78	−0.79	2.25	1.45	−1.70	0.035
Affirmative Passive	3.25	2.29	−2.11	3.79	2.54	−4.57	ns
Negative Passive	2.54	1.82	−2.58	3.09	1.69	−3.45	ns
Relative	2.44	1.93	−2.53	2.35	1.32	−3.59	ns
Dative	2.08	1.37	−5.47	1.44	1.35	−4.23	ns
**Receptive Vocabulary (LQ PPVT-R)**	82.15	10.74	−1.19	81.39	16.61	−1.24	ns
Grammatical production level (GASS) *	4.16	0.61	-	4.62	0.56	-	0.002
**Expressive Vocabulary for high-frequency words (One-Word Picture Vocabulary Test)**	14.04	5.84	−0.47	12.42	3.73	−0.52	ns
**Expressive Vocabulary for low-frequency words (One-Word Picture Vocabulary Test)**	32.11	6.76	−0.72	32.69	4.56	−1.21	ns
**NVIQ**	101.35	14.83	-	100.38	16.63	-	ns
**VIQ**	89.36	26.55	-	90.21	20.18	-	ns
**TIQ**	94.18	13.52	-	90.33	20.59	-	ns
**ADOS Comparison Score**	5.36	1.38	-	5.20	1.35	-	ns

* *p* < 0.05. Abbreviations: SD: standard deviation; LQ: Lexical Quotient; NVIQ: Non-verbal Intelligence Quotient; VIQ: Verbal Intelligence Quotient; TIQ: Total Intelligence Quotient.

**Table 2:** Comparison between males and females with receptive disorder and between males and females without receptive disorder.

	**Males with** **Receptive** **Disorder** **(*n* = 31)**	**Females with** **Receptive** **Disorder** **(*n* = 17)**	***p***	**Males without** **Receptive** **Disorder** **(*n* = 19)**	**Females without** **Receptive** **Disorder** **(*n* = 9)**	***p***
**Age**	80.71 (11.43)	84.59 (8.52)	ns	68.53 (10.65)	70.11 (14.23)	ns
Grammatical Comprehension (TCGB, z score) *	−4.4 (2.43)	−7.02 (3.57)	0.014	−0.54 (0.61)	−0.52 (0.66)	ns
**Receptive Vocabulary (PPVT-R. LQ)**	80.09 (10.77)	76.22 (11.43)	ns	84.67 (10.45)	86.56 (19.89)	ns
Grammatical production (GASS level) *	4.00 (0.63)	4.47 (0.62)	0.019	4.39 (0.52)	4.89 (0.33)	0.028
**Expressive Vocabulary for high-frequency words (One-Word Picture Vocabulary Test, z score)**	−1.02 (1.46)	−0.99 (1.23)	ns	0.41 (0.86)	0.36 (0.88)	ns
**Expressive Vocabulary for low-frequency words (One-Word Picture Vocabulary Test, z score)**	−1.06 (1.2)	−1.64 (0.82)	ns	−0.16 (1.14)	−0.40 (0.47)	ns
**NVIQ**	98.77 (15.43)	93.59 (15.15)	ns	105.42 (13.22)	113.22 (11.01)	ns
**ADOS Comparison Score**	5.44 (1.42)	5.38 (1.31)	ns	5.24 (1.35)	4.89 (1.45)	ns

* *p* < 0.05. Abbreviations: LQ: Lexical Quotient; NVIQ: Non-verbal Intelligence Quotient.

## Data Availability

Data created or analyzed during this study is available upon request from the corresponding author.

## Supplementary Materials

The detailed description of TCGB and GASS can be downloaded at: https://www.mdpi.com/article/10.3390/jcm12154923/s1, Table S1: The Test of Grammatical Comprehension for Children TCGB; Table S2: Grid of Analysis of Spontaneous Speech GASS.
